# Addressing the challenges of climate-driven community-led resettlement and site expansion: knowledge sharing, storytelling, healing, and collaborative coalition building

**DOI:** 10.1007/s13412-021-00695-0

**Published:** 2021-06-02

**Authors:** Julie Maldonado, Itzel Flores Castillo Wang, Fred Eningowuk, Lesley Iaukea, Aranzazu Lascurain, Heather Lazrus, Chief Albert Naquin, JR Naquin, Kukuya Margarita Nogueras-Vidal, Kristina Peterson, Isabel Rivera-Collazo, M. Kalani Souza, Mark Stege, Bill Thomas

**Affiliations:** 1 Environmental Studies Program, 4312 Bren Hall, UC Santa Barbara, Santa Barbara, CA 93106-4160 USA; 2 Livelihoods Knowledge Exchange Network, Lexington, KY USA; 3 Shishmaref, Shishmaref, AK USA; 4 University of Hawai‘i at Mānoa, Honolulu, HI USA; 5 Southeast Climate Adaptation Science Center, Raleigh, NC USA; 6 National Center for Atmospheric Research, Boulder, CO USA; 7 Isle de Jean Charles Biloxi-Chitimacha-Choctaw Indians of Louisiana, Parish, Terrebonne, LA USA; 8 Comunidad Tribu Yuke de Jayuya, Jayuya, Puerto Rico USA; 9 Lowlander Center, Gray, LA USA; 10 University of California-San Diego, San Diego, CA USA; 11 Olohana Foundation, National Disaster Training Preparedness Center/FEMA, University of Hawai‘i, Honolulu, HI USA; 12 Maloelap Atol Local Government, Majuro, Marshall Islands; 13 National Oceanic and Atmospheric Administration, Washington, DC USA

**Keywords:** Community-led resettlement, Site expansion, Indigenous knowledge, Climate adaptation, Coalition building, Knowledge sharing

## Abstract

Presently coastal areas globally are becoming unviable, with people no longer able to maintain livelihoods and settlements due to, for example, increasing floods, storm surges, coastal erosion, and sea level rise, yet there exist significant policy obstacles and practical and regulatory challenges to community-led and community-wide responses. For many receiving support only at the individual level for relocation or other adaptive responses, individual and community harm is perpetuated through the loss of culture and identity incurred through forced assimilation policies. Often, challenges dealt to frontline communities are founded on centuries of injustices. Can these challenges of both norms and policies be addressed? Can we develop socially, culturally, environmentally, and economically just sustainable adaptation processes that supports community responses, maintenance and evolution of traditions, and rejuvenates regenerative life-supporting ecosystems? This article brings together Indigenous community leaders, knowledge-holders, and allied collaborators from Louisiana, Hawai‘i, Alaska, Borikén/Puerto Rico, and the Marshall Islands, to share their stories and lived experiences of the relocation and other adaptive challenges in their homelands and territories, the obstacles posed by the state or regional governments in community adaptation efforts, ideas for transforming the research paradigm from expecting communities to answer scientific questions to having scientists address community priorities, and the healing processes that communities are employing. The contributors are connected through the Rising Voices Center for Indigenous and Earth Sciences, which brings together Indigenous, tribal, and community leaders, atmospheric, social, biological, and ecological scientists, students, educators, and other experts, and facilitates intercultural, relational-based approaches for understanding and adapting to extreme weather and climate events, climate variability, and climate change.

“May we do the good work for our people, may we do the good work in the memory of our grandparents, and for the new memories of our great-grandchildren.”

– Rev. M. Kalani Souza, Olohana Foundation, June 1, 2020,

Virtual Rising Voices 8, Community Relocation/Site Expansion Panel Session

Presently coastal areas globally are becoming unviable, with people no longer able to maintain livelihoods and settlements due to, for example, increasing floods, storm surges, coastal erosion, and sea level rise. Whole population groups are being displaced or pushed out of places; up to 13.1 million people in the USA are projected to relocate from coastlines before 2100 (Hauer et al. 2016). The scale of displacements increases as tipping points and thresholds in the climate system are crossed, particularly challenges on the intensity, frequency, and duration of extreme weather events. Increased emissions, temperature rise, and melting glaciers are resulting in worst-case scenarios already being exceeded (Hansen et al. 2015). Climate change has exacerbated the physical risks and threats frontline communities are exposed to every day. Many individuals and communities are already making adaptation decisions, including relocation, to reduce their exposure to climate change impacts now and in the coming decades (USGCRP 2018). For some communities facing extreme climate-driven risks, relocation is an adaptation method necessary for survival, yet there are significant policy obstacles and practical and regulatory challenges to community-wide and -led actions. For many receiving support only at the individual level for relocation or other adaptive responses, individual and community harm is perpetuated through, for example, loss of culture and identity incurred through forced assimilation policies. Challenges dealt to frontline communities are often founded on centuries of injustices. The current moment in time has allowed us to recommit ourselves to understanding these deep seated historical injustices. Can these challenges of both norms and policies be addressed? Can we develop socially, culturally, environmentally, and economically just sustainable adaptation processes that supports community responses, maintenance, and evolution of traditions, and rejuvenates regenerative life-supporting ecosystems, including food and water sources, green infrastructure, and future growth? This paper offers a path forward to address these challenges and develop just sustainable adaptation processes.

How, and especially by whom, relocation plans are developed will have substantial impacts on people’s lives and entire ecological systems, which are intimately interlinked. These impacts are particularly critical for individuals in frontline communities who are enduring disproportionate hardship from climate and environmental risks and have historically suffered under decisions made by people outside their communities (Maldonado et al. 2020), and on ecological systems already degraded by unsustainable development and extractive practices.

State and federal government officials have spent years working to preemptively relocate more than a dozen small coastal communities in Alaska and Louisiana that are disappearing into the rising sea (GAO 2020). An estimated 2300 Puerto Rican families displaced by Hurricane Maria are still looking for permanent housing (Silva 2018). The extreme disruptions in our climate system and migration patterns calls for new innovations, concepts, tools, strategies, and initiatives embraced by leaders shaping governance decisions and influencing cultural shifts. How do we build flexibility and resilience in governance and institutional responses to community-led and –based relocation that focus on the sociocultural opportunity to assist the entire collective community? How do we integrate the interwoven priorities that climate-driven migration call for in terms of long-range planning tools to support anticipatory and reflexive strategies that recognize the demands and potentially positive outcomes? How do people relocate with their cultures intact? How do communities work to restore their whole community, including economic development, strategies to maintain and preserve culture, and provide safe, viable housing? Is that possible? It is certainly not going to be possible if community discussions of these themes are not part of the political and institutional conversation (Maldonado et al. 2020).

This article brings together Indigenous community leaders, knowledge-holders, and allied collaborators from Louisiana, Hawai‘i, Alaska, Borikén/Puerto Rico, and the Marshall Islands to share their stories and lived experiences of climate adaptation, including relocation, challenges in their homelands and territories, the obstacles posed by the state or regional governments in community adaptation efforts, ideas for transforming the research paradigm from expecting communities to answer scientific questions to having scientists address community priorities, and the healing processes that communities are employing. The article focuses on three questions in particular: (1) What challenges are communities experiencing in efforts to implement adaptation responses, including relocation? (2) What obstacles are posed by the state or regional government and/or scientific institutions in implementing community adaptation priorities?; and (3) What are community practices in healing from the challenges and obstacles in the climate adaptation process?

What follows is a focused discussion on community’s climate adaptation responses, including the challenges for communities working on community-led and -wide relocation, resettlement, or expanding into a new or ancestral site when adapting in place is no longer a viable option. The perspectives shared in this article are not the authors’ alone. For many of the author team, the insights shared are from the Indigenous and local knowledge they hold based on lived experience and passed down over many generations. The following is part of an on-going, emerging dialog from a decade of collaboration through the Rising Voices Center for Indigenous and Earth Sciences^1^, which brings together Indigenous, tribal, and community leaders, atmospheric, social, biological, and ecological scientists, students, educators, and other experts, and facilitates intercultural, relational-based approaches for understanding and adapting to extreme weather and climate events, climate variability, and climate change. During the novel coronavirus pandemic and a time of physical distancing, the Rising Voices Center transformed its annual in-person workshop into a series of online, thematic-focused dialogs over several months. 2 Continuing to build from the work of the Rising Voices Community Relocation and Site Expansion Working Group, one of the sessions in the series was a panel discussion on climate-driven displacement and community relocation. The following dialog emerged from this discussion, as well as drawing on nearly a decade of collaboration and multi-generational knowledge and wisdom.

## What challenges are communities experiencing in efforts to implement adaptation responses, including relocation?

“It is a very complicated issue and it is systemic; it extends well beyond the current challenges of climate change. It’s completely tangled with power relationships, with identity issues, with political relationships, with economic hardships and constraints… We have to first think of where are we, who we are, and where we’re going.” – Dr. Isabel Rivera-Collazo

Across the varying contexts, from the Pacific Islands to Louisiana to the Caribbean Islands to Alaska, community land acquisition, within the specific context of the history of lands and traditions, emerges to the forefront of community priorities when faced with climate-driven displacement. Particularly in the context of increasing climate-driven risks, land stewardship and access can mean safety, as well as increasing food security, self-determination, and sovereignty to stay connected and identify with one’s Indigenous roots. Coastal fishing communities in Boriké, for example, face coastal erosion that threatens their homes and livelihoods; communities living in the mountains are threatened by rain and landslides that can disrupt their pathways and access to food and resources. The island is experiencing increasing sea level rise, more frequent intense rainfall events and associated coastal flooding, and saltwater intrusion, along with storm surges and high energy wave action that will likely cause coastlines in Puerto Rico to be submerged or greatly reduced in extent (Bhardwaj et al. 2018; Ezcurra and Rivera-Collazo 2018; Gould et al. 2018; Hopkinson et al. 2008; Mercado-Irizarry 2017).

Elder Nogueras-Vidal and Dr. Isabel Rivera-Collazo, both from Boriké, explain that for the Indigenous communities in Boriké, Bieke, and Culebra (Puerto Rico), it is important to signal the challenging circumstances around relocation or site expansion^3^ as part of climate change adaptation. Indigeneity in Boriké has to be contextualized in historical processes of identity disenfranchisement, and the complex geography of a multiethnic archipelago. Similar to Barbuda (Boger et al. 2019), traditional household tenure in Boriké includes continued occupation on the same location along generations of the same family. This traditional practice however means that many often do not hold legal title to their traditional lands and lack the acquisition power or financial means to either purchase the land they live on or other land where they could relocate to if needed. This reality also impacts their possibility of rebuilding a house after a disaster, as land tenure and title are required for insurance and permit processes.

In addition, Elder Nogueras-Vidal emphasizes that the Indigenous People of Boriké live in pocket communities scattered around the island, where they practice their traditional culture wherever they are able to do so. COVID-19 has dramatically impacted these practices, given that many of their community members are elders at-risk who need to remain safe from infection. Governmental restrictions on gatherings and travel between communities have also disrupted their traditional practices, and technology (including phones, computers, and internet) are not widely available. They are also unable to thrive because of the lack of adequate land ownership, which is needed in order to develop a sustainable and self-sufficient way of life and economy for and by the community. In recent years, particularly after Hurricane Maria, they have experienced land grabs by powerful groups and big corporations, such as Bitcoin (Bowles 2018), and the growing tourism industry which is developing projects within their communities, disrupting and destroying sacred lands and the people’s ancestral legacy. Furthermore, pharmaceutical and other chemical companies have been discharging toxic waste in the air, water, and land for decades, causing major health issues in the island’s population including cancer, diabetes, asthma, birth defects, emotional traumas, and other issues in the surrounding communities.

Boriké (Puerto Rico) is at very high risk due to climate change and the Islands’ geographical location (Keellings and Hernández Ayala 2019). Their people presently find themselves in a constant risk of impact due to more intense and sudden or unexpected atmospheric events throughout the year, such as hurricanes. Cumulative and on-going climate impacts compound the effects of landslides, loss of mountain roads, regular flooding, loss of houses, permanent mold exposure within damaged houses, and coastal erosion, among others (Seara et al. 2020; Butterworth et al. 2017; Ferré et al. 2019; Ramos-Scharrón et al. 2020; Barreto-Orta et al. 2019; Rivera-Collazo 2020). In addition, regular strong earthquakes, thousands of which have been experienced since January 2020, contribute to uncertainty, livelihood insecurity, and overall stress, severely impacting community members’ mental and physical health. There is a real need for the co-development of a mitigation plan that can enable the people to survive the onset of disasters such as earthquakes, hurricanes, and tsunamis, as well as land-loss due to sea level rise, taking into consideration their needs and priorities as Indigenous Peoples of Boriké (Barreto-Orta et al. 2019; Butterworth et al. 2017; Ferré et al. 2019; Keellings and Ayala 2019; Ramos-Scharrón et al. 2020; Rivera-Collazo 2020; Seara et al. 2020).

Similarly, the Isle de Jean Charles Biloxi-Chitimacha-Choctaw Indians of Louisiana (IDJC-BCC) are prioritizing obtaining land where their community can safely resettle and maintain their cultural integrity, especially given the ecological transformation and impacts that climate change is having on their lands, including flooding due to hurricanes, storms, and sea level rise, which already has and continues to displace families. Sea level rise is increasing the frequency and extent of extreme flooding associated with coastal storms (USGCRP 2017), and hurricane-induced rainfall rates are projected to increase, resulting in increased flooding along the Gulf Coast, including Louisiana (Frankson and Kunkel 2017). Ninety-eight percent of the Island’s 33,000 acres of landmass has vanished due to relative sea level rise, erosion, oil and gas infrastructure, and levee development (Maldonado 2019). The initiative to purchase land has been in the works with the Louisiana government for nearly two decades (IDJC-BCC 2020).

Most recently, the IDJC Tribal leadership’s continued efforts in planning for resettlement included engaging with the State of Louisiana. The State’s proposal for the US Housing and Urban National Disaster Resilience Competition in 2015 included the Tribe’s Resettlement Plan, focused on cultural preservation and reuniting the Tribe, including those who had already been forced to move (Lowlander Center 2015). In 2016, the State was awarded the funding, including $48 million to build the Tribe’s envisioned resettlement (Comardelle 2020). After working to locate the plot of land that the IDJC Tribal Council designated as a safe place where their entire community—not just individual families—could relocate to, the State’s “slow response and improper execution” of the grant, and their last significant amendment (No. 5; see Louisiana Office of Community Development-Disaster Recovery Unit 2019) to the resettlement “made it clear that the IDJC Tribe was no longer a beneficiary of nor involved in the grant process” (Comardelle 2020). As Chief Albert Naquin put it, “The State hijacked [their] project.” The IDJC Tribal Council and many IDJC Tribal citizens have witnessed the State abandon its commitment to support the Tribe’s distinct vision articulated in the funded application and in doing so, undermined the Tribe’s efforts to preserve their cultural heritage, improve their economic conditions, enhance their cultural resilience, and protect their Tribal rights through the resettlement process. The State’s vision for resettlement is proving to be assimilationist, with potential disastrous results in moving people from the coast without preserving and strengthening social relationships and distinct traditional lifeways that have been strained by this imposed crisis of land loss (IDJC-BCC 2019; see also Dermansky 2019a, b; Jessee 2020; Yeoman 2020).

Without protection from increasing land loss, repeat flooding, and storm surges, there has been no other alternative for the Tribe but to move north. The Tribal leadership is looking into other funding sources that may be more supportive of the Tribe’s vision and priorities for their resettlement, to uphold the Tribe as rights-holders with historical ties to their ancestral territory and each other and committed to cultural continuity and future generations of IDJC Tribal family, knowledge, and ways of life. The IDJC Tribe’s cultural survival depends on it (IDJC-BCC 2019). Even with the ecological transformation and challenges experienced through the resettlement process, the Tribe continues to advocate for and work to perpetuate their culture and preserve their place (Comardelle 2020).

For the village of Shishmaref, Alaska, located approximately 30-miles south of the Arctic Circle and home to approximately 600 Inupiat people, Elder Fred Eningowuk articulated how climate change is impacting the land and ocean, from which they sustain life. As Mr. Eningowuk articulated, “The ocean and land are our garden and supermarket and how we survived for thousands of years” (Rising Voices 2015). In recent winters, the temperature did not reach −30^o^F as it used to; the community has noticed many environmental changes, such as earlier spring ice breakup, later ice buildup, changes in how slush builds on the shores to form a natural wall for the community, melting permafrost, less snow each year, less berries because of changing snow patterns, and erosion because of lack of accumulated snow (Rising Voices 2015). They have also witnessed unusual low tides, as well as high water without storms, signaling the need for localized data on sea level rise (Rising Voices 2016). As a community, they voted to relocate in 1973, 2002, and again in 2016 (Shishmaref Interagency Planning Work Group, n.d.), before a major storm and subsequent flooding leads to further damage, including loss of human life. While they have worked on the relocation process for nearing two decades, they are trying to adapt to climate change, such as harvesting spring mammals 1 month earlier than before.

Considering, in part, a community’s constraints to obtain services in-place once they are ear-marked for relocation (Marino 2015), the word “relocation” was removed through a local election when the Native Village of Shishmaref decided to move. They changed the wording to “site expansion” to enable more projects for the community. For example, since they removed the word “relocation” and used the word “site expansion,” their community has gained a new fuel tank farm, a new health clinic, an expansion to their public school, worked on getting paved roads, a new washeteria, and repairing and upgrading the airport. Nonetheless, they still need money to build an additional seawall to protect their island from erosion, and the community remains, for the most part, without running water, impacting residents’ health. The clinic, school, and washeteria are the only services with running water. During winter, they can drink ice water and during rainfall season, they have that water supply available. However, these water resources are not available year-round; in their home, they have to work for their water (Rising Voices 2018).

Relatedly, in Oceania, Marshallese Islanders and their government have been concerned about climate change and its implications for long-term adaptation. The Marshall Islands and surrounding ecosystem have already experienced warmer air temperatures, warmer sea surface temperatures that lead to coral bleaching, serious flooding, and increasing sea level rise, among other climate changes (RMI 2014). According to Maloelap Atoll Councilman Mark Stege, an atoll adaptation specialist from the Marshall Islands, they have two horizons for adaptation strategies. They are presently in the short-term horizon, which includes coastal and flood protection infrastructure, climate proofing, and strengthening water and food security as well as job resilience. The concern is that many of the smaller islands are already low-lying atolls and have no elevation for communities to move back or up to, thus making it evident that their first line of defense, the short-term mechanisms currently in place, may be their last defense as well.

Relatedly, in the Central Pacific Ocean, Marshallese and their government have been concerned about climate change and its existential implications for their atoll nation (Letman 2018). According to Councilman Stege, atolls are unique in that they have “habitability thresholds” that are extremely finite (Stege 2018). “Our first line of defense is our last line,” he says. A partnership with the Unitarian Universalist Service Committee (UUSC) is assisting Councilman Stege and his constituents in the remote atoll community of Maloelap to conduct climate variability assessments needed to understand what their best options are for adaptation. Some of the strategies that they have been testing and putting into practice are based on accrued experience and the traditional ecological knowledge they hold. Councilman Stege weaves in support with developing elevation models and wave-driven flooding scenarios on those models, searching for approaches on how to bring together his communities’ traditional ecological knowledge with the Western-oriented approaches. The UUSC is allowing them to determine their own climate research agenda in which they are “looking for ways for the traditional and the modern to come together and inform [their] future adaptation needs…trying to test out the idea of identity” and ultimately producing identity-enhancing flood risk models.

## What obstacles are posed by the state or regional government and/or scientific institutions in implementing community adaptation priorities?

The colonial relationship between the USA and its occupied nations and territories has had tremendous impact on communities’ food security and food sovereignty (Whyte 2018). Currently, communities have been forced to move away from their self-sufficient food supply system and instead engage in the globalized market of food supply systems (Rivera-Collazo et al. 2018). This becomes extreme in the context of occupied territories such as Boriké, where, as Dr. Rivera-Collazo and Elder Nogueras-Vidal explained, they are only allowed to hire ships that sail under the American flag. When those ships’ navigation is affected by hurricanes and storms, the community are left with limited access to resources. This was the case of the S.S. El Faro, which sank on October 1, 2015, after being impacted by the eye of Hurricane Joaquin while en route to Puerto Rico (Bell and Kirtman 2019). Communities in Boriké and throughout the US Caribbean, forced to rely on imported food and goods and services, are left critically exposed to climate-related disruptions in transportation systems (Gould et al. 2018; Rivera 2018). This calls for changing the way in which the government operates, such as considering antiquated federal laws like the Merchant Marine Act of 1920, known as the Jones Act (Maritime Law Center, n.d.), which requires shipping between the US ports to be conducted by the US-flag ships (Legal Information Institute, n.d.). Presently, there are only a fraction of the number of the US ships that were around when the Act was established.

Governmental, economic, and other institutions pose particular challenges to traditional, tribal, and indigenous communities in their efforts for a just support system for climate adaptation. Of particular relevance is the issue of land grabs and the need to secure land. The IDJC Tribal Council lost the support and land towards which they had been working in part because of the scarcity of available land and continuous shifts in the price of properties. Multinational oil and gas corporations and private land developers contemporarily purchase vast quantities of land on higher ground just north of Isle de Jean Charles, leaving the Tribe and other coastal communities with few options (Maldonado 2019). In their case, the price of the property that the Tribe had planned for escalated tremendously from the first time they had looked at it to the time that it was purchased by the State. Land needs to be properly secured from the first moment it is considered, and kept intact and ready to be utilized by communities, giving them a choice of where they can go rather than government entities choosing who and how many can receive help, and directing the community on where to go. One distinct challenge is that the government is not a trusted entity with many communities at risk, in particular their experiences with colonial-driven structural violence.

As experienced by the Isle de Jean Charles Biloxi-Chitimacha-Choctaw Indians of Louisiana, the governance systems developed to aid communities in relocation and other adaptive processes are often marred with political, institutional and administrative barriers, interpretations, and rules with jargon, confusing and unclear language, and which do not include community input in the process or consider climate change as an attributable variable. This in turn causes harm to communities when applying for grants, aid, etc., as they may apply without full understanding of the “fine print,” and/or can be misguided due to the language used to describe the objective and requirements of these policies. As Dr. Kristina Peterson, co-founder of the non-profit Lowlander Center and long-time collaborator with tribes and other frontline communities in coastal Louisiana, articulated, “[W]e have a new kind of Manifest Destiny going on that’s called ‘managed retreat,’ and it’s managed by those who didn’t take any regard for who or what was in place or how to respect people’s lifeways or to respect people’s cultures and background and we have to circumvent that kind of mentality.”

The challenge lies within the context of the United States’ history and its mission to assimilate Native People and countless communities and people that have come to form a part of its territory (e.g., Puerto Rico). Part of that history is the taking away of land, language, and culture, which in turn causes communities to become dependent on the food, language, and culture that is given to them or become essentially isolated. It is critical for Indigenous People and other frontline community members to be in decision-making spaces and conversations. As Dr. Lesley Iaukea, a Native Hawaiian educator expressed, “Going into these meetings, sometimes the people are not thinking about the culture. They’re thinking about the economic purposes, they’re not even thinking about equality…[but] it is okay to sit there and talk about how it would affect us, where we come from, describe our side of it, to humanize it.”

It is also critical to understand that frontline communities are not homogenous and are not all in the same position or context. For example, the Marshall Islands are an independent and democratically elected atoll nation, with a level of self-determination within their communities that reflects their highly traditional land tenure system that has endured a 200-year history of colonialism from Spain, Germany, Japan, and the USA relatively intact. This history and their current concerns with climate change have led them to truly look at the complexities of their institutions, their situation, and their identity. The challenge for them is not a matter of relocation just yet; rather, the challenge lies in building the social resilience networks and partnerships needed both internally and externally to see their plans through. As Councilman Stege articulated, he does not know “if relocation is something on the tip of our tongues just yet. Science will say otherwise but I think there’s more to it than getting the climate models and wave models figured out. If you’re looking at a 30, 50 year timeframe, there’s time. And I think it’s okay to take it at the pace needed by the community.”

For Shishmaref, they have been working with the Denali Commission on their expansion efforts, and made attempts to get land through the Bering Land Bridge National Preserve. Until the expansion is accomplished and they can move to the mainland, a quarry will be utilized for protection of their land. Shishmaref has 5, 10, and 15-year strategic management plans, which were developed by doing door-to-door surveys and interviewing all members residing in Shishmaref; these plans are to be used between now and the time when they expand. There is a community coalition that includes the City of Shishmaref, Shishmaref Native Corporation, City Council, Tribal Council, Elders, and Youth, which holds monthly meetings to continue moving their work forward. Their community supports each other, which is important for getting things done. They are also part of a coalition with other Alaska Native villages working together on their projects. This coalition building and work and solidarity efforts are part of the healing in moving forward through the site expansion process.

## What are community practices in healing from the challenges and obstacles in the climate adaptation process?

As Rev. M. Kalani Souza, Native Hawaiian knowledge-holder, Hawaiian practitioner, and cross-cultural conflict resolution facilitator, expressed, “we’re only going forward together… [and it] is going to take real strength of character, of purpose, of country, of identity,” but we have to remember that this is not the first time these processes have occurred. Though Western European history may not go back that far, each other’s ancestral roots show that migration has happened before and it has been and continues to be consequential to the planet and to us. We must continue to humanize these processes and create a nexus and intersection of understanding among all of us.

Reflecting on the quote famously attributed to Albert Einstein—“The intuitive mind is a sacred gift and the rational mind is a faithful servant. We have created a society that honors the servant and has forgotten the gift”—Rev. Souza pointed to the fact that in today’s society, we reward the rational servant, those with the doctorate degrees, while those with the intuitive gift are disregarded as being less. Elder Nogueras-Vidal expressed that we can create our own realities; it is a practice she has adopted in her own life, dreaming of the way things should be and in turn ensuring that those dreams become a reality. Their elders and ancestors before them have had this intuitive gift, have passed it down to them, and if they begin to utilize it and tap into this spiritual connection, it will assist in guiding their path. Healing will come from their Indigenous roots and from bringing back their knowledge and soul, and reuniting them into the healing process so that they may continue dreaming, forming connections, persevering, and thriving. For the Native Village of Shishmaref, the island life is an integral part of their way of life. To help mitigate the culture loss, in the community’s vision statement in their strategic management plan, they do not focus on relocation but rather on the positive outcomes: “Shishmaref is a safe and resilient community. We want to be a viable community that respects and honors our Inupiat culture and traditional values. We will work together and with partners to develop projects and policies to protect our residents, infrastructure, natural environment, and subsistence resources. We will preserve and enhance our community for us and future generations” (HDR with RIM First People, Cox, Eningowuk 2016).

Interwoven within this healing is the trauma that has become part of the colonial history and the impacts of the federal government’s policies and actions on their communities. Chief Albert Naquin challenged that healing is not a problem for their community, but rather the problem is how to heal the federal government that continues to complicate matters; the Isle de Jean Charles Biloxi-Chitimacha-Choctaw Indians of Louisiana and other Tribes that are not federally recognized have had to live through the government telling them they could not attend school because they were “Indians” and now telling them they are not “Indian” enough and having to prove their Indigeneity before being able to access benefits provided by the government. Part of this experience, as Dr. Rivera-Collazo reflected, includes dealing with learning a history that has erased Indigenous roots and identity, a colonial process that continues to perpetrate the idea that all Indigenous people are gone and that claiming any connection to Indigenous roots is invalid, or if one claims Indigeneity, they must look a certain way to be able to do so. History books are written by those who hold the most power and who choose to filter history and recreate a reality that erases Indigenous identities; as such, “the healing begins by acknowledging that we are still here.”

It is critically important, Dr. Iaukea articulated, to accept that intergenerational trauma is real and address the implications this has had and continues to have within their communities. The process of alleviating and healing this pain and trauma, for her, is about learning their language and not using it simply as a tool, but understanding it and using it as a way to understand their ancestors more. It is about holding onto their stories and places, practicing traditions, learning why their ancestors did what they did, and of talking with their elders, and listening to them and understanding them. We must collectively learn from the past to inform the future, and to know what questions to ask in the present (Rising Voices 2016). Mr. Stege explained that learning their history in the Marshall Islands and putting it into the context of what the future may hold for them and their islands is important. Climate science is needed to help answer questions such as which islands will hold ground and stay as long as possible, but the history of their communities will also play an important role in ensuring that these processes will be self-determining and healing through the process of adaptation.

## Where do we go from here?

“The data and funding are all important issues but without the human aspect it goes nowhere…so as we move forward we must keep the aspect of humanity in the forefront of our discussions along with understanding the importance of spirituality because they connect us with the past, the future and the present, and those that are around us and those that are not...embrace all of those complexities…it’s not easy but we do it because…everybody behind us depends on what we do.” – Mr. Bill Thomas

The dialog above focused on issues of land, the access to and acquisition of it, and of communication and language challenges, including the legal implications of these topics. There is an important shift, however, in the focus of including the humanizing aspects, and the important role of knowing who they are, where they came from, and where they are going will play in the processes of adaptation. As Mr. Bill Thomas, a Native Hawaiian scientist and the Senior Advisor for Islands, Indigenous, and International Issues at the National Oceanic and Atmospheric Administration Office for Coastal Management, expressed, “actually speaking not just from our place but from our hearts on these issues.” The scientific community cannot be averse to including such conversations of identity and culture into climate science and policy adaptation discussions, while also acknowledging the challenges of claiming Indigenous identity in a society that continues to perpetuate that Indigenous People do not exist.

Elder Nogueras-Vidal shared how communities are working to prioritize their needs. The elderly and the children are presently at “high risk” due to poor health conditions and depression, among other threats. They are working towards providing support for and also uplifting each other through maintaining their connection with each other, and they are teaching the people to empower themselves through their relationship with the Great Spirit, which is central to the Indigenous way of life. They are in need of land in order to thrive as a people. With land, they can create a settlement to build community resilience, sustainability, and economic viability.

Each community has unique histories, traditions, priorities, and needs to be considered before any plan for relocation, site expansion, adaptation, or mitigation to climate change can be implemented (Thomas et al. 2019). Considering the emerging contexts under which communities are working to realize their priorities for climate change adaptation actions, a critical question becomes, how do we humanize the policies and process of adaptation, including relocation? While terms such as forced migration, relocation, and managed retreat are used to describe management plans to aid communities, the communities themselves, their experiences, input, history, language, and identity are often left out of and off of the official discussion table.

There is a distinct need to prioritize inclusion of cultures, histories, and knowledge systems in adaptation processes, including supporting and upholding the visionary insights and guidance of Indigenous communities (see also Rights and Resources Initiative 2019). Indigenous ancestors have moved from different lands to new ones and they have continued their culture and ways to thrive. As such, in the context of climate-driven migration or displacement, it is critical to support communities in continuing their cultures, traditions, and practices in new places. These unique community-driven decisions make communities more resilient. If our policy intentions are preserving human lives and community diversity, then this approach is imperative.

One example to learn from is the relocation process experienced by people from the Island Nation of Tokelau, which was hit by a hurricane in the 1960s and four islands became uninhabitable. The residents were forced to move and were taken in by the government of Aotearoa/New Zealand. Dr. Lesley Iaukea, a Native Hawaiian educator and scientist, who has long worked and engaged with those who experienced this relocation, articulated how they were put to work, learned English, and used as second-class citizens. In the process what became erased in their next generation was the language, traditions, and the religious or spirituality aspect. In 2000, the elders realized that of the 70% that moved to New Zealand, about half of them could no longer speak their native language or even hold a sentence in Tokelauan. They started a grassroots movement to bring back the language and culture. This movement featured members of the community opening up their garages to have church services and gatherings, and as people began noticing these spaces featured the Tokelauan language, the movement grew and within five years they saw the number of language speakers rise. By 2014, they began to introduce this movement into the school system and New Zealand incorporated 14 native languages into their public education system. From being an island-nation that had to forcibly move, and then moving and not being able to speak their language, the elders held it together, and about 20 years later, they had the language at the school for future generations to learn their language. They found a way to keep their culture and roots, and even bring that and introduce it into their new place.

Adapting to a new location often results in a fundamental loss of identity and the forced assimilation that can be a consequence of this process. To prevent and mitigate this attack on identity, it is critical to recognize that communities are not only bound geographically, but through relationships and practices (Marino et al. 2019). Community-led resettlement requires including the communities’ voices and input in all decisions. It is imperative that the Tribal and other community leaders and organizers who have spent a generation and more working on such efforts are the ones guiding their process to hold community’s rights and sovereignty intact. Tribes and Indigenous Peoples, regardless of federal recognition status, have a unique position at the decision-making table as collective rights-holders. The “table” is actually theirs, with outside scientists, agencies, and policymakers being the invitees, through established relationships following protocol of the people and place with respect, reciprocity, and responsibility. Community leaders and organizers should be engaged from visioning and planning through implementation, and beyond relocation or site expansion to ensure success in the aftermath of physical resettlement (Marino et al. 2019). The loss of land does not mean the loss of rights. This is especially critical in the challenges of administrative law that is often inflexible when it comes to Tribes and Indigenous Peoples. This calls for establishing mutual trust and respect between all partners involved in the adaptation process; prioritizing in the scientific community—connection, transparency, and respect; and focusing on a relational and relationship-based rather than transactional approach, including consideration of nonmaterial elements such as histories, practices, and ways of life that are essential in thriving communities (Rising Voices 2019; also Maldonado et al. 2020).

Proactive planning to meet communities’ priorities and needs must be done at the level of rights, sovereignty, self-determination, protections, security, and concepts of integration (see for example, UUSC and Rising Voices-Working Group 2021). A rights-based approach to address climate change works to change the current dynamic, to add flexibility and resilience to local government responses. This approach creates a shift away from policies that are against something, such as viewing migrants as a burden, and moves policies in a direction of being for something, a positive perspective eliciting pro-action, empowerment, pride, and dignity in the face of much uncertainty. Such an approach can support to adapt and thrive in the context of the increasing climate crisis, choosing dignity over victimization.

We often focus on discussing the limitations of land, Bill Thomas reflected, but we forget that though land sometimes separates us, we have all also seen how oceans naturally unite us. We all have had a history of migration and it has happened because of the dreams, the visions, and the visionaries who came before us. We must be leaders and control and create our own narratives. We must look at the importance of storytelling and understand the context in which those stories have been told in order to understand where we came from and where we are going, and more importantly, we need to hold on to our intuitive side. We must make time to stop and ask questions, and listen to our intuition.

## Data Availability

All reports from where this material is drawn can be found here, https://risingvoices.ucar.edu/annual-workshops.

## Footnotes

1 Rising Voices is co-administered by the National Center for Atmospheric Research (NCAR), which is managed by the University Corporation for Atmospheric Research (UCAR), and the Livelihoods Knowledge Exchange Network (LiKEN), in partnership with the National Oceanic and Atmospheric Administration's Office for Coastal Management (NOAA-OCM) and Haskell Indian Nations University, among many more partners. Funding support for the program comes primarily from the UCAR Office of Diversity, Equity, and Inclusion and from the NOAA-OCM Grant Award NA18NOS4730188.

2 Throughout the article, when a quote does not have a direct citation it is from the Virtual Rising Voices 8, Rising Voices Community Relocation/Site Expansion Panel Session, which took place in June 2020. For more information, https://risingvoices.ucar.edu/annual-workshops.

3 “Site expansion” is language that has emerged specifically around and from Kigiqtamiut residents in Shishmaref, Alaska. Site expansion is used to indicate that the tribe maintains control over their island, even in the case of new infrastructure and residency within a new site on the mainland. For more about the implications and importance of this, see, for example, Marino et al. 2019.
